# A Comparative Study on the Effects of “Honey and Fenugreek” with “Fenugreek” on the Breastfeeding Success: A Randomized Trial

**DOI:** 10.1155/2022/6048280

**Published:** 2022-06-23

**Authors:** Masoumeh Simbar, Soheila Nazarpour, Faraz Mojab, Farahnaz Kholosi Badr, Mobina Khorrami, Zahra Jafari Torkamani, Hamid Alavi-Majd

**Affiliations:** ^1^Midwifery and Reproductive Health Research Center, Shahid Beheshti University of Medical Sciences, Tehran, Iran; ^2^Department of Midwifery and Reproductive Health, School of Nursing and Midwifery, Shahid Beheshti University of Medical Sciences, Tehran, Iran; ^3^Department of Midwifery, Chalous Branch, Islamic Azad University, Chalous, Iran; ^4^Department of Pharmacognosy, School of Pharmacy, Shahid Beheshti University of Medical Sciences, Tehran, Iran; ^5^North Health Center, Shahid Beheshti University of Medical Sciences, Tehran, Iran; ^6^Department of Biostatistics, School of Paramedicine, Shahid Beheshti University of Medical Sciences, Tehran, Iran

## Abstract

**Objective:**

Herbal galactagogues are usually recommended to be sweetened with honey. Despite the high nutritious value of honey for lactating women and extensive studies on the effects of herbal galactagogues, no investigation was made to compare galactagogue effects of using herbal galactagogues with and without honey so far. The present study aimed to compare the effect of a combination of “honey and fenugreek” with “fenugreek” on breastfeeding success (BFS).

**Methods:**

This study is a triple-blind randomized clinical trial on 75 breastfeeding mothers referred to a Tehran-Iran health center. The participants were randomly divided into two intervention (fenugreek and honey users) and control (fenugreek users) groups. These women had infants of 1–5 months and had exclusive breastfeeding practice. Tools for data collection were (1) the personal and maternal-infant information, (2) the breastfeeding success, and (3) the complications questionnaires. Participants received 30 liquid drops, three times daily for four weeks. The BFS questionnaire was completed twice, before and 4 weeks after the intervention. The CONSORT checklist was followed.

**Results:**

Before and after comparison showed no significant difference in the BFS score in the “fenugreek” group, while there was a significant increase in the BFS score in the “honey and fenugreek” group (*P*=0.035). Between groups, comparison demonstrated a significantly higher score of BFS in the intervention group than in the control group (*P*=0.023). There were no significant differences between the two groups regarding the possible complications.

**Conclusion:**

The combination of honey with fenugreek showed a significant effect on BFS, while there was no improvement in BFS after fenugreek only uses. Trial registration: the study was approved in the Iranian registry of clinical trials with IRCT ID: IRCT20120122008801N23 on 2019-12-30 (https://www.irct.ir/).

## 1. Introduction

The World Health Organization emphasizes breastfeeding as a natural infant feeding method. The World Health Organization and UNICEF recommend exclusive breastfeeding of infants without any further fluids or solids for the first six months of life [1]. Promoting exclusive breastfeeding is critical to achieve many of the sustainable development goals by 2030, both in rich and poor countries, because of its protective effects against noncommunicable diseases (such as breast cancer, diabetes, overweight, and obesity) and so improving maternal and infants' health [2]. However, the rate of exclusive breastfeeding in low- and middle-income countries is only 37 percent [2].

Although breastfeeding is associated with many positive health consequences, accepting exclusive breastfeeding as the preferred method of infant feeding is challenging [3]. Worldwide, maternal perception of insufficient milk production is the most common reported reason of mothers for the early discontinuation of breastfeeding [4]. Many interventions are recommended to increase breast milk production and the prevalence of exclusive breastfeeding such as psychological support, relaxation, and nutritional interventions [5]. However, many mothers seek to increase their milk production with pharmacological or nonpharmacological natural galactagogues [3, 6]. Natural galactagogues are usually medicinal herbs or other foods that stimulate milk production [7]. There is a wide range of natural galactagogues for mothers who do not choose pharmacological alternatives for increasing milk production in many cultures around the world [8].

Mothers from different cultural and ethnic backgrounds often choose different methods according to their tradition and experience. Some of the more popular herbs are fenugreek (Trigonella foenum-graecum), fennel (Foeniculum vulgare), Ginger (Zingiber officinale), Ecoflora (Cnicus benedictus), Plectranthus amboinicus (Coleus amboinicus Lour), asparagus (Asparagus racemosus), Anise (Pimpinella anisum), Sunflower (Silybum marianum), Barley (Hordeum vulgare), Moringa (Moringa oleifera), Galega (Galega officinalis), Marsh-mallow (Althaea officinalis), Common Nettle (Urtica dioica), black caraway (Nigella sativa), basil (Ocimum basilicum), cumin (Cuminum cyminum), grape, and coffee [7, 8]. More than 400 plants have been introduced as a galactagogue in the world [9]. Despite the long-term use of the natural galactagogue, scientific evaluation has not been performed to prove the clinical efficacy of most of them [8]. Among these herbs, fenugreek is one of the most widely used galactagogues [10, 11]. Fenugreek (Trigonella foenum L.) is a dicotyledonous plant [12] that is historically used for its anti-inflammatory and antioxidative effects [13] as well as for decreasing the severity of labor pain and dysmenorrhea [14, 15]. Also, fenugreek seeds are used as a galactagogue [13] and are usually recommended for breastfeeding enhancement in Iranian traditional medicine [6]. However, herbal medicines, including herbal galactagogues such as fenugreek that are commonly and traditionally used by the general population, are not necessarily effective and require scientific evidence-based documents to prove their effectiveness.

Herbal medicines and galactagogues are usually recommended to be sweetened with honey [16]. Honey is produced by bees (*Apis mellifera* L.) from the nectar of plants or from secretions of living parts of plants which the bees collect, transform by combining with specific substances of their own, deposit, dehydrate, store, and leave in the honeycomb to ripen and mature [17]. Honey has a very long history of low-risk food use. It is often consumed alone, as a spread, or may be mixed with a wide range of other foods. Honey is a natural substance known for its therapeutic effects from ancient times. Flavonoids and phenolic acids play a key role in the high antioxidant and anti-inflammatory effects of honey [18]. Honey is a supersaturated sugar solution. Its composition is complex and variable, and it contains at least 181 different substances. These substances can mainly be divided into two groups: the major compounds such as the monosaccharide (glucose and fructose) and the minor compounds including amino acids, enzymes, vitamins and minerals, and polyphenols. The ingredients of honey have been reported to exert antioxidant, antimicrobial, anti-inflammatory, antiproliferative, anticancer, and antimetastatic effects [19–21].

Honey is traditionally used in combination with herbal galactagogues [22]. There is only a study showing the effectiveness of sule honey consumption in increasing milk production of working mothers [23]. Besides, a study showed an increase in milk production after using honey as a dietary supplement for lactating cows [24]. Despite the high nutritious value of honey for all, especially for puerperal and lactating women, and a huge number of comparative studies on the effects of herbal galactagogues, no investigation was made to compare galactagogue effects of using herbal galactagogues with and without honey so far [25]. Therefore, this study hypothesized that consuming a combination of honey and fenugreek is more effective than consuming fenugreek alone on breastfeeding success. The present study aimed to compare the effect of “a combination of honey and fenugreek” with “fenugreek” on breastfeeding success.

## 2. Methods

### 2.1. Study Design

It was a triple-blind randomized parallel clinical trial to compare the effect of “a combination of honey and fenugreek” with “fenugreek” on breastfeeding success.

This study was performed on 80 breastfeeding mothers who were referred for controlling their infants' growth and development to a public health center from the health network system in Tehran-Iran. This sort of health center is providing a range of healthcare services including monitoring of infants' growth and development and breastfeeding counseling.

### 2.2. The Participants

The study participants were breastfeeding mothers who were referred to a public health center with the following inclusion criteria: age 18 to 38 years, having 1- to 5-month infant, having exclusive breastfeeding, no history of known medical and psychological diseases, nonsmoking, with normal single fetus pregnancy, normal term delivery, and maternal body mass index (19.8–26 kg/m^2^), and were seeking advice for a natural galactagogue. They had no breast problems such as abscesses, little, or sagging nipples, and were not taking milk-interfering medications, any synthetic galactagogues, or antibiotics. The infants were mature at birth and weighed between 2.5 and 4 kg and did not have any abnormalities, diseases, or nutritional problems.

The exclusion criteria were as follows: incidence of any mental or physical illness during the study that interferes with breastfeeding such as hepatitis, cancer, and breast problems; use of other herbal and chemical drugs that increase milk during the intervention, use of dopamine antagonists (domperidone, metoclopramide, risperidone, and phenothiazine), cessation of breastfeeding for any reason, and unwillingness to continue the intervention. Considering 6 months in the definition of exclusive breastfeeding, neonates with a maximum age of 5 months were included in the study to be included in the definition of breastfeeding after one month of intervention.

### 2.3. Sampling

The sample size was obtained using the following equation in each group, as it is suggested to be used for comparing two means in the randomized clinical trials [26]:

(1) $$\begin{array}{c} n\geq 2\frac{{\left(z_{\alpha /2}+z_{\beta }\right)}^{2}\sigma^{2}}{{\left(\mu_{1}-\mu_{2}\right)}^{2}},\\ \alpha =0.05\Rightarrow z_{\alpha /2}=1.96,\\ \beta =0.20\Rightarrow z_{\beta }=0.85,\\ 1-\beta =0.80,\\ \frac{\left(\mu_{1}-\mu_{2}\right)}{\sigma }=0.65,\\ n=2{\left(1.96+0.85\right)}^{2}{\left(\frac{1}{0.65}\right)}^{2}=37. \end{array}$$

The effect size was considered 0.65 obtained from a similar study by Vahdat and Vahdat study [27] on the effect of fenugreek seed on the signs of breast milk sufficiency, and considering the 10% drop in patients in each group, 41 samples were calculated for the recruitment of the samples.

The participants with inclusion criteria were consecutively selected and randomly assigned to two groups of intervention and control. Using the Excel software randomization option, the eligible women were given the A and B coded drop bottles of “fenugreek” and “honey and fenugreek.”

### 2.4. Blinding Process

The “honey and fenugreek” and “fenugreek” drops were made by the pharmacognosist (FM). He coded the bottles of the medicines as A and B. The researchers and the participants as well as the statistician were not informed about the codes, and decoding was performed until the end of the data analysis.

### 2.5. Tools for Data Collection

Two questionnaires were used for data collection.

1. A personal and maternal-infant information questionnaire to collect information about “demographic, maternal-infant, and breastfeeding condition.” This questionnaire included questions about demographic questions including age, level of education, employment of the participants and their husbands, and income status. Also, a history of pregnancy (including gravid, para, and abortion, wanting the pregnancy, stress, and anxiety before and during pregnancy), the history of delivery (including the type of delivery, anesthesia, place of delivery, sex of the baby, the weight of neonate at birth, and the history of breastfeeding (initiation time of breastfeeding, skin-to-skin contact, and family support)).
2. Breastfeeding status was asked by questions about the average frequency of breastfeeding in 24 hours, at night, and the baby's restlessness.
3. A questionnaire to assess “breastfeeding success” including 21 items, which were mainly extracted from the instructions of the Ministry of Health of Iran for successful breastfeeding [28] and also an extensive related literature review. The items of the questionnaire are shown in Table 1.
4. The content validity of the questionnaire was assessed by 10 midwifery and reproductive health experts from faculty members of Shahid Beheshti University of Medical Sciences. The experts assessed the questionnaire in terms of grammar, wording, and item allocation (qualitative content validity). Also, the assessment was based on the Waltz & Bausell content validity index (CVI) [29]; the experts scored the relevancy, clarity, and simplicity of each item through a 4-point Likert scale, and the CVI for each item was calculated by dividing the number of experts who scored items a 3 or 4 by the total number of experts. The statement was accepted if the CVI was ≥0.79 [30]; the necessity of the items was assessed through a 3-point rating scale: (i) not necessary; (ii) useful, but not essential; and (iii) essential. Following the experts' assessments, a content validity ratio (CVR) for the total scale was computed. According to Lawshe, an acceptable CVR value for 10 experts was 0.62 [30].
5. Scale-level content validity index (S-CVI) and scale-level content validity ratio (S-CVR) were computed by calculating the mean of CVI and CVR values, where S-CVI >0.70 is considered acceptable [29]. The validity was confirmed by S-CVI = 0.79 and S-CVR = 0.84, respectively.
6. The reliability of the questionnaire was evaluated by internal consistency and stability assessment. Internal consistency was shown by Cronbach's alpha 0.79. The stability of the questionnaire was assessed by the test-retest method by the completion of the questionnaires by 15 lactating women twice at a two-week interval before initiation of the study. Then, the stability was shown by the Pearson correlation = 0.73 of test-retest scores.
7. This questionnaire was designed to measure the success rate of breastfeeding by signs of breast milk sufficiency, as the outcome variable.
8. Scoring procedure: the final questionnaire contains 21 items, which are scored 1 to 5, for never, rarely, sometimes, often, and always choices, respectively. Items 19 to 21 are scored reversely. The minimum and maximum scores are 21 to 105. The total score was calculated by summing the scores of 21 items. The higher scores show more success in breastfeeding.
9. The questionnaire was filled up twice through an interview by the trained researchers with the participant, once before intervention and then 4 weeks after intervention.
10. The complications questionnaire that was also filled up by the participants after 4 weeks of intervention to assess maternal or infant's possible complications of the intervention. The questionnaire contained 6 items about the occurrence of possible complications during the intervention including maternal or infant skin complications, maternal nausea and vomiting, maternal gastrointestinal problems (indigestion, constipation, and diarrhea), maternal headache and vertigo, sleep disorders including drowsiness in mother or infant, and infants' gastrointestinal problems (vomiting, constipation, and diarrhea).

The questionnaires were filled up by the trained researchers with the participants twice, through a face-to-face interview once before the intervention and then through a telephone interview 4 weeks after the intervention.

### 2.6. Material

The “fenugreek” and the “honey and fenugreek” liquid drops were prepared by the Pharmacognosy Department of Shahid Beheshti University of Medical Science. The two medicines “honey and fenugreek” and “fenugreek” were used as the natural galactagogue for the intervention and control groups.

#### 2.6.1. The Fenugreek

The origin of fenugreek in this study was Damghan region of Iran. Fenugreek seeds were purchased from the herbal market in Tehran Iran in May 2020. The expiration date was of three years as seeds remain edible 3 years after harvest as long as they are kept in an airtight container and away from sunlight. The oral route is the conventional and most widely used route for the administration of dosage forms. Among different formulations, syrups are best because they impart a sweet taste to the formulation and are thus advantageous over bitter drugs [31]. Fenugreek is slightly sweet and pleasantly bitter [32]. Therefore, we prepared liquid drops from fenugreek seeds, and both groups were informed about consuming the liquid drops with a sweet taste.

To make fenugreek extract, fenugreek seeds were first powdered and soaked in ethanol 96°, three times during the night. The solvents were evaporated (below 40°*C*), and then, the concentrated solvent was used as the extract. The yield of extraction was 15%. The concentration of the extract was 8.4 mg in 1 mL solvent.

#### 2.6.2. The Combination of Fenugreek and Honey

For the intervention group, the extract was added to 70% honey syrup, and for the control group, the extract was added to the hydro-aqueous solution. The honey used in this study was prepared from the Khansar-Iran region of Iran. The natural honey was purchased from Rayehe Market in Tehran-Iran. These drugs were prepared and manufactured in similar bottles and coded the by pharmacognosist of the study in the Pharmacognosy Department of the School of Pharmacy of Shahid Beheshti University of Medical Sciences.

### 2.7. The Procedure of the Study

At the beginning of the study and after a detailed explanation about the study, the participants with the inclusion criteria completed the written consent. Then, before the intervention, “the personal and maternal-infant information” and “breastfeeding success” questionnaires were completed through a face-to-face interview by the trained researchers with the participant. The women who were willing to use natural galactagogues were randomly devoted to two groups of the “honey and fenugreek” and “fenugreek.” Both groups were prescribed a daily dose of 30 liquid drops in three times per day (every 8 hours), for four weeks. The participants were in contact with the researchers during the four weeks and the researchers answer any questions. Then, the “breastfeeding success” and “complications” questionnaires were completed by the researchers through a telephone interview with the participants at the end of four weeks of intervention. The sampling started June 2020 and ended in February 2021.

### 2.8. Data Analysis

Data were analyzed using SPSS 24 statistical software (SPSS Inc., Chicago, IL, USA). Independent and paired t-tests were used to compare quantitative variables with normal distribution, and the Mann–Whitney U test was used for quantitative variables with non-normal distribution. Kolmogorov–Smirnov test was used to test the normality of the data distribution (*P* > 0.05). Before and after comparisons were performed by using paired *t*-test. Qualitative variables were compared by the chi-squared test. To compare the effect of two types of drops in the two groups, an analysis of covariance (ANCOVA) was used. In this test, the BFS score before the intervention was considered a covariate. The primary outcome of this study was the breastfeeding success score that was compared before and after the intervention in the two groups. The complications were also compared between the two groups. The *P*-values less than 0.05 were considered significant.

### 2.9. Ethics

The study was approved by the Ethics Committee of Shahid Beheshti University of Medical Sciences, with the code “IR.SBMU.PHARMACY.REC.1398.225.” Written informed consent was obtained from all participants. The study was assessed and approved in the Iranian registry of clinical trials with IRCT ID: IRCT20120122008801N23 and the IRCT Code 44309 on 2019-12-30 (https://www.irct.ir).

## 3. Results

**Figure 1:**
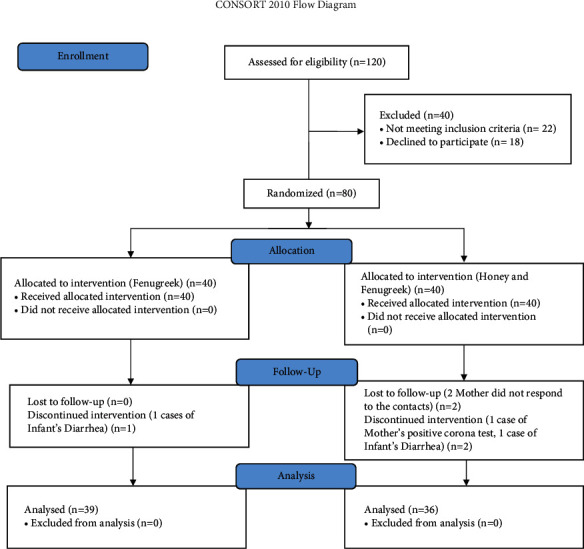
The CONSORT (consolidated standards of reporting trials) flow chart.

Eighty women with the eligibility criteria were recruited for the study. During the four weeks of the study, 5 out of 80 participants were excluded from the study including 1 woman of the “fenugreek” group and 4 women of the “fenugreek and honey” group. Finally, 75 patients, including 39 and 36 women in the “fenugreek” and “honey and fenugreek” groups, respectively, completed the clinical trial (Figure 1).

The finding showed no significant difference between the two groups of the study regarding personal characteristics, before the intervention (Table 2).

Results also demonstrated no significant difference between the two groups respecting the breastfeeding condition and the infants' weight, length, and head circumference before intervention (Table 3). There was no significant difference between the two groups in assessing the success rate of breastfeeding before the intervention (*P*=0.534).

Intragroup comparison in the “fenugreek” group showed no significant difference in BFS before and after intervention (paired *T*-test; *P*=0.388), while there was a significantly higher BFS score in the “fenugreek and honey” group after the intervention compared to before the intervention (*P*=0.035) (Table 4)

Between-groups comparison by ANCOVA showed a significantly higher BFS score of the “honey and fenugreek” group than the “fenugreek” group (*P*=0.023) (Table 4).

The finding demonstrated no significant difference between groups of “fenugreek” and “honey and fenugreek” with respect to the possible complications (Table 5). The reported possible maternal complications were nausea and vomiting (1 case in the “honey and fenugreek” group), gastrointestinal complications including pain, indigestion, constipation, and diarrhea (2 cases in the “fenugreek” and 1 case in “honey and fenugreek” group). The possible complications in infants included diarrhea (6 cases in the “fenugreek” and 1 case in the “honey and fenugreek” group). In general, the complications were not significantly different between the two groups. Consumption of grip mixer (grip mixer syrup contains ingredients such as sugar and mint that are used for infants to relieve bloating, abdominal pain, and nausea) in infants of the two groups during the intervention did not show a significant difference (*P*=0.168).

## 4. Discussion

The present study showed that a combination of “honey and fenugreek” improves BFS. This was the first clinical trial to compare the effects of a “combination of honey” with “fenugreek” on BFS. Several review and descriptive studies are reported on using honey as a galactagogue [33]. In Iranian traditional medicine, honey is suggested to be used by lactating women as a galactagogue [25]. Honey is also recommended for the treatment of insufficient breast milk by Indian tribal communities [22]. Also, the addition of honey as a sweetener to herbal galactagogues especially herbal teas is also recommended in the traditional medicine of many countries [22, 34–37]. For example, in the Baiga tribe in Euphorbia, lactating women use hirta (Dudhai) fresh plant water with honey as the galactagogue [22]. Also, in the Thiruvananthapuram region, milk yam (Ipomoea digitata L.) and tuber powder with cow's milk with honey are used as a galactagogue [38]. Use of honey with Nigella seed [36, 39–41] and fennel [40] are also recommended for increasing milk production. So, this question was raised that honey could have effects on the results and it can be a galactagogue itself. Despite a huge number of studies that show significant effects of natural galactagogues, there is only a study by Baroroh that showed the effectiveness of sule honey consumption in increasing milk production for working mothers [23]. An animal study also showed an increase in milk production after using honey as a dietary supplement for lactating cows [24].

So, we demonstrated the combination of “honey and fenugreek” is significantly more effective than “fenugreek” on the signs of breast milk adequacy as the criteria for BFS.

This is the first report on the positive effect of honey on BFS. Evidence-based research shows that honey acts through a modulatory road of multiple signaling pathways and molecular targets. It may interfere with multiple targets in cell signaling pathways such as induction of caspases in apoptosis; stimulation of TNF-*α*, IL-1*β*, IFN-*γ*, IFNGR1, p53, and immune cells; inhibition of cell proliferation; cell cycle arrest; inhibition of lipoprotein oxidation, IL-1, IL-10, COX-2, LOXs, and PGE2; and modulation of other diverse targets. This results in triggering the amelioration of antioxidant, antimutagenic, anti-inflammatory, immunoregulatory, and estrogenic responses to abate different types of diseases. It seems further research is needed to establish the possible mechanisms involved in the lactogenic possible effects of honey [42]. More experimental trials are also intended to validate the authenticity of honey either alone or as adjuvant therapy for breast milk insufficiency.

No significant difference was found between total scores of BFS before and after the intervention in the “fenugreek” group. Reeder and colleagues reported also no significant difference between the milk volume of mothers taking fenugreek and mothers receiving a placebo [43]. However, this result is not consistent with the results of some other studies [10, 11, 27, 44–46]. For instance, Khan et al. indicated that consumption of fenugreek significantly increased the amount of the produced breast milk (11.11, CI 95% 6.77, 15.46) versus placebo by a network meta-analysis on four studies to test the significance of the galactagogue effect of fenugreek administrated to lactating women versus placebo or control groups [14]. Therefore, it seems further evidence is necessary to support claims of the galactagogue effect of fenugreek. Foong et al. in a Cochrane base systemic review on oral galactagogues for increasing breast milk production concluded that due to extremely limited, very low certainty evidence, we do not know whether the galactagogues have any effect on infant weight and milk volume in mothers with healthy, term infants. Besides, because of the substantial heterogeneity of the studies, imprecision of measurements, and incomplete reporting, the magnitude of the effect is not ascertained [7]. Additionally, the difference could be attributed to the dose and the delivery system of the drugs. The drug delivery system (tablets, capsules, syrups, etc.) enables the release of the active pharmaceutical ingredient to achieving a desired therapeutic response [47]. The delivery system in our study was the liquid drops, and in the study of Reeder et al., it was a capsule, while in most other studies where fenugreek was effective, it was used as an herbal tea [10, 45, 46] or fenugreek water [11]. It seems that consuming fenugreek as an herbal tea or water leads to more milk volume and more frequent breastfeeding, but its effect on milk composition is unknown. Besides, the fenugreek failed to increase the BFS and the dosage could be an effective factor for this finding. We used different similar studies to find out the possible effective dosage of fenugreek. So, future systemic review studies are necessary to clarify the effects of fenugreek on breastmilk.

Although fenugreek consumption could not increase the success score of breastfeeding in the present study, the addition of honey to fenugreek showed a significant effect on BFS. Therefore, it seems that honey can be used to improve the effect of fenugreek or even other galactagogues. We chose Fenugreek as the control as it is one of the most common medicinal herbs used by lactating mothers around the world including in Iran [7]. Besides, fenugreek is traditionally recommended as a galactagogue in Iran. In this study, it was suggested to the participants who were seeking a natural galactagogue. Fenugreek is listed as GRAS (generally regarded as safe) by the U.S. Food and Drug Administration [48, 49].

There was no significant difference between “honey and fenugreek” “fenugreek” groups with respect to the possible complications. These observed complications during 4 weeks of the intervention may or may not be attributed to these herbal remedies. Although the two groups were not significantly different regarding diarrhea complications, 6 cases of diarrhea in the “fenugreek” group may be due to the laxative effect of fenugreek [13] that seems to be limited by adding honey as there was only one case of diarrhea in the “fenugreek and honey” group. Honey is demonstrated to be used in the treatment of diarrhea in traditional medicine [50]. It is also demonstrated that honey improves the health of the gut by acting as a prebiotic agent [51]. Daily intake of honey as food could easily reach 100 g in some individuals, a dose far higher than is likely to be achieved when honey is consumed in therapeutic forms [52].

In the present study, we measured the BFS as the indicator for measuring the outcome of the study. The BFS questionnaire contains 21 items, which are the signs of breast milk adequacy. This was a valid and reliable questionnaire to assess the BFS, and it was used in a previous study [53]. Experimental studies on improving breastfeeding measure different indicators as the outcomes such as infants' growth indicators (e.g., weight, length, head, chest, and arm circumference of infants) newborn wet and defection diaper counts, and mother's prolactin level [54]. For instance, Ghasemi et al. measured the infant growth parameters including weight, length, and head circumference of infants [10]; Mathew et al. weighed the infants [46]; Abdou and Fathey measured mothers' serum prolactin levels [44]; Srinivas et al. used infants' anthropometric indices as well as mothers' serum prolactin levels [49]; Ravi and Joseph measured infant's weight and frequency of urination [11]; and El Sakka et al. study measured breast milk volume by manual breast pumping [45]. Similar to our study, Vahdat and Vahdat assessed the signs of breast milk adequacy by a daily follow-up form, as well as measuring infants' growth parameters and the number of wet diapers and duration of lactation [27]. We measured the BFS by a valid and reliable questionnaire including 21 items to assess the signs of adequacy as the indicator for the BFS.

### 4.1. Limitation of the Study

In this study, we intended to measure and compare the anthropometric characteristics including weight, head circumference, and height of infants before and after the intervention; however, we could not measure these variables as the follow-up outcome after the intervention, due to corona-related limitations and the absence of mothers after the intervention.

Also, breastfeeding may lead to hypoglycemia and we did not ask about the participants' sugar intake during breastfeeding. However, we asked about the complications during the intervention such as drowsiness or headache, which could be symptoms of hypoglycemia and were not different between the two groups.

In this study, the effectiveness of two possible galactagogues including a “combination of honey and fenugreek” with “fenugreek alone” was compared. The intervention group could be “honey alone”; however, since the subjects of the study were selected from those who were seeking for natural galactagogues and, meanwhile, the healthcare providers in the health centers routinely suggest fenugreek and fennel teas as the galactagogue, the “combination of honey and fenugreek” was compared with the “fenugreek” as the control of the study.

In this study, serum prolactin levels were not measured, while in the studies related to the effect of galactagogues on breastfeeding, prolactin levels can be a more precise outcome measurement.

In addition, longer follow-up outcomes such as neonatal anthropometric indices are recommended to evaluate the effect of galactagogues on neonatal growth rate.

In this study, we used Khansar honey, and it should be noted that the composition of honey to some extent depends on the flowers, the geographical area, and the season, and in this study, the composition of honey was not exactly clarified.

## 5. Conclusion

Fenugreek consumption could not increase the BFS; however, the addition of fenugreek to honey showed a significant effect on BFS. Therefore, it seems that honey can be used to improve the effect of fenugreek or even other herbal galactagogues.

The combination of “honey with fenugreek” is a natural galactagogue that can improve the success of breastfeeding and is recommended for promoting breastfeeding practice.

## 6. Relevance to Clinical Practice

Many breastfeeding women ask for natural galactagogue when they attend for postpartum care or their infants' growth and development monitoring. Due to the global trend of people toward herbal medicines, especially in traditional Eastern societies, including Iran, healthcare providers usually recommend herbal and traditional medicines such as fennel and fenugreek in the form of teas, syrups, and drops. In many cases, these are recommended to be sweetened with honey. This study showed that honey with fenugreek can be suggested and consumed as a galactagogue.

## Figures and Tables

**Table 1:** Breastfeeding success questionnaire (BSQ-21).

Please fill in the form based on the information from the last four weeks		Always	Often	Sometimes	Seldom	Never
1	For the past four weeks, your baby has been sleeping comfortably after each feeding					
2	Every time you breastfeed, there were signs of satiety (falling asleep) for your baby.					
3	You have breastfed your baby every time she/he cries					
4	If, after breastfeeding, the baby seeks for the breast again, you breastfeed her/him again					
5	The baby has taken both breasts without any problems.					
6	The infant had regular suctioning for at least 10 minutes at a time					
7	The infant is breastfed from two breasts at a time					
8	You feel full in your breasts before breastfeeding					
9	After breastfeeding, you feel that your breasts are softer					
10	The infant had defecation 2–4 times a day with sufficient volume (more than a spot)					
11	The baby has urinated at least 6 times a day					
12	When an infant sucks on the breast, you hear the sound of regular sucking and swallowing					
13	She/he has given up breastfeeding every time she completes breastfeeding					
14	By the end of breastfeeding, the infant has relaxed and calmed down					
15	Each lactation lasts about 15–20 minutes					
16	The infant is fed every 2–3 hours					
17	The baby wakes up only once during the night to breastfeed					
18	You feel like your breastfeeding has been good for the past four weeks					
19	For the past four weeks, you have been feeding your baby a food other than your own					
20	The infant appears hungry after most of the feeding					
21	Over the past four weeks, your baby has become restless					

**Table 2:** Comparison between personal characteristics of two groups of the study.

Characteristics	Fenugreek group (*n* = 39)	Honey and fenugreekgroup (*n* = 36)	Test	
	*n* (%)/ mean ± SD	*n* (%)/ mean ± SD	*Independent t*-test	
Age (years)	32.46 ± 7.00	31.75 ± 4.97	*P*=0.616	
				*Mann-Whitney test*
Number of pregnancies (gravidity)	1.54 ± 0.68	1.42 ± 0.50	*Z* = −0.529	*P*=0.597
Number of deliveries (parity)	1.44 ± 0.64	1.47 ± 0.70	*Z* = −0.012	*P*=0.990
Number of abortions	0.08 ± 0.27	0.14 ± 0.49	*Z* = −0.169	*P*=0.866
Number of live children	0.41 ± 0.59	0.33 ± 0.53	*Z* = −0.534	*P*=0.593

				*Chi-squared test/Fisher'sexact test*
Educational level	Under diploma and diploma	7 (18.0)	7 (19.4)	*P*=0.173
	Associate diploma	6 (15.4)	2 (5.6)	
	Bachelor	14 (35.9)	21 (58.3)	
	Master and doctorate	12 (30.8)	6 (16.7)	
Job	Housewife	28 (71.8)	23 (63.9)	*P*=0.463
	Employed	11 (28.2)	13 (36.1)	
Income^*∗*^	Inadequate	3 (7.7)	0 (0.0)	*P*=0.089
	Adequate	36 (92.3)	36 (100.0)	
Wanted pregnancy^*∗*^	Yes	35 (89.7)	35 (97.2)	*P*=0.195
	No	4 (10.3)	1 (2.8)	
Stress and anxiety	Yes	3 (7.7)	6 (16.7)	*P*=0.232
	No	36 (92.3)	30 (83.3)	
Type of delivery	NVD^†^	12 (30.8)	8 (22.2)	*P*=0.403
	CS^‡^	27 (69.2)	28 (77.8)	
Place of childbirth^*∗*^	Hospital	38 (97.4)	36 (100.0)	*P*=0.333
	Home	1 (2.6)	0 (00.0)	
Gender of baby	Girl	24 (61.5)	21 (58.3)	*P*=0.777
	Boy	15 (38.5)	15 (41.7)	

^*∗*^ Fisher's exact test was used as the expected frequencies are lesser than 5. ^†^NVD: normal vaginal delivery; ^‡^CS: cesarean section.

**Table 3:** Comparison between maternal-infant characteristics and breastfeeding condition of two groups of the study before intervention.

Infants' anthropometric characteristicsand breastfeeding condition		Fenugreekgroup (*n* = 39)	Honey and fenugreekgroup (*n* = 36)	Test
		Mean ± SD	Mean ± SD	Independent*t*-test
Birthweight (kg)		3.21 ± 0.37	3.15 ± 0.33	*P*=0.397
Weight of infant (kg)		6.12 ± 1.61	5.98 ± 1.98	*P*=0.739
Length of infant (cm)		61.84 ± 6.03	61.13 ± 7.67	*P*=0.674
Head circumference of infant (cm)		39.39 ± 3.27	39.82 ± 3.39	*P*=0.604

		*n* (%)	*n* (%)	*Chi-square test/Fisher's exact test*
Breastfeeding Initiation time	Immediately	26 (66.7)	20 (55.6)	*P*=0.408
	After 1 hour	7 (17.9)	12 (33.3)	
	After 2-3 hours	3 (7.7)	3 (8.3)	
	After 4–6 hours	1 (2.6)	1 (2.8)	
	More than 6 hours	2 (5.1)	0 (0.0)	
Nipple care	Yes	36 (92.3)	34 (94.4)	*P*=0.711
	No	3 (7.7)	2 (5.6)	
Family support of breastfeeding	Yes	37 (94.9)	33 (91.7)	*P*=0.578
	No	2 (5.1)	3 (8.3)	
Skin-to-skin contact	Yes	38 (97.4)	34 (94.4)	*P*=0.509
	No	1 (2.6)	2 (5.6)	

**Table 4:** Intragroup and between-groups comparison of breastfeeding success before and after the intervention in the two “fenugreek” and “fenugreek and honey” groups.

Groups	*Intragroup comparison*		
	Time	Mean ± SD	*Paired t-test (P*)
Fenugreek (*n* = 39)	Before intervention	77.46 ± 7.30	0.388
	After intervention	76.33 ± 8.08	

Honey and fenugreek (*n* = 36)	Before intervention	72.97 ± 6.72	0.035
	After intervention	77.80 ± 12.19	
	*Between-groups comparison*		
		Mean ± SD	*ANCOVA (P*)
Fenugreek (*n* = 39)		75.53 ± 1.64	0.023
Honey and fenugreek (*n* = 36)		78.67 ± 1.71	

**Table 5:** Comparing the possible complications after four weeks of “fenugreek” and “fenugreek and honey” use.

Complications	Fenugreek group *n* (%)	Honey and fenugreek group *n* (%)	Chi-squared test (*P*)
Maternal or infant skin complications	0 (0.0)	0 (0.0)	—
Maternal nausea and vomiting	0 (0.0)	1 (2.8)	0.295
Maternal gastrointestinal problems^†^	2 (5.1)	1 (2.8)	0.604
Maternal headache and vertigo	0 (0.0)	0 (0.0)	—
Sleep problem in mother or infant	0 (0.0)	0 (0.0)	—
Vomiting of infant	0 (0.0)	0 (0.0)	—
Constipation of infant	0 (0.0)	0 (0.0)	—
Diarrhea of infant	6 (15.4)	1 (2.8)	0.061

^†^Gastrointestinal problems include pain, indigestion, constipation, and diarrhea.

## Data Availability

The datasets used and/or analyzed during the current study are available from the corresponding author on reasonable request.

## Abbreviations

BFS: Breastfeeding success
UNICEF: United Nations International Children's Emergency Fund
CVI: Content validity index
CVR: Content validity ratio
S-CVI: Scale-level content validity index
S-CVR: Scale-level content validity ratio
SPSS: Statistical Package for the Social Sciences
ANCOVA: Analysis of covariance
IRCT: Iranian registry of clinical trials.

## Supplementary Materials

Supplementary Materials The supplementary files include a CONSORT 2010 checklist of information and a personal and demographic information questionnaire form.
