# Supplementary material for: A Comparative Study on the Effects of “Honey and Fenugreek” with “Fenugreek” on the Breastfeeding Success: A Randomized Trial
Source: Evid Based Complement Alternat Med. 2022 Jun 23;2022:6048280. doi: 10.1155/2022/6048280 (PMC9246572; doi:10.1155/2022/6048280)
Supplement: Supplementary Materials — The supplementary files include a CONSORT 2010 checklist of information and a personal and demographic information questionnaire form. [file 6048280.f1.zip › 6048280.f1/Demographic and personal information form.docx]

**Demographic and personal information form**

Dear mother, the information that you provide to us by completing this questionnaire is completely confidential, please help us in achieving the purpose of the study with your correct answers.

Completion date of the questionnaire: / /

Part One: Demographic Questions

1. Age ............ years

2. Education level:

 illiterate  undergraduate  diploma  master diploma  bachelor  master and doctorate

3. Employment status:

Housewife Home job Worker Employee freelance job

4. Spouse's employment status:

Unemployed Worker Employee آزادFree Job Type of Job ..........

5. Economic status of the family:

Less than enough  As much as enough  I can save

Part II: Pregnancy history

6. Number of pregnancies: ....

7. Number of deliveries: .........

8. Number of abortions: ......

9. Number of other living children.........

10. Recent type of pregnancy:  Wanted  Unwanted

11. Do I have a history of diabetes before and during pregnancy?

Yes No

12. Do I have a history of stress and anxiety before and during pregnancy?

Yes  No

13. Place of delivery: Home hospital

14. Gender of the baby: girl  boy

15. Birth weight: ............. grams

Part III: History of breastfeeding

16. How long after birth did you start breastfeeding your baby?

Immediately One hour later  2-3 hours later -4-4 hours later  More than 6 hours later

17. Do you take care of your nipples to prevent sores and cracks? Yes  No

18. Do you receive support from family members in child care? Yes  No

19. Do you receive support from family members while breastfeeding? Yes  No

20. Do you make skin-to-skin contact with your baby during breastfeeding? Yes  No

**Infant’s growth checklist**

| Index | First time (before intervention) | ~~After intervention~~ |
| --- | --- | --- |
| Infant weight (g) |  |  |
| Infant height (cm) |  |  |
| Infant head circumference (Cm) |  |  |

**)**
